# A Three-Dimensional Finite-Element Model of a Human Dry Skull for Bone-Conduction Hearing

**DOI:** 10.1155/2014/519429

**Published:** 2014-08-27

**Authors:** Namkeun Kim, You Chang, Stefan Stenfelt

**Affiliations:** Department of Clinical and Experimental Medicine, Linköping University, 58185 Linköping, Sweden

## Abstract

A three-dimensional finite-element (FE) model of a human dry skull was devised for simulation of human bone-conduction (BC) hearing. Although a dry skull is a simplification of the real complex human skull, such model is valuable for understanding basic BC hearing processes. For validation of the model, the mechanical point impedance of the skull as well as the acceleration of the ipsilateral and contralateral cochlear bone was computed and compared to experimental results. Simulation results showed reasonable consistency between the mechanical point impedance and the experimental measurements when Young's modulus for skull and polyurethane was set to be 7.3 GPa and 1 MPa with 0.01 and 0.1 loss factors at 1 kHz, respectively. Moreover, the acceleration in the medial-lateral direction showed the best correspondence with the published experimental data, whereas the acceleration in the inferior-superior direction showed the largest discrepancy. However, the results were reasonable considering that different geometries were used for the 3D FE skull and the skull used in the published experimental study. The dry skull model is a first step for understanding BC hearing mechanism in a human head and simulation results can be used to predict vibration pattern of the bone surrounding the middle and inner ear during BC stimulation.

## 1. Introduction

The human auditory nerve is connected to the microstructure called “organ of Corti (OC)” in the cochlea. The OC is located on the basilar membrane (BM). Therefore, the motion of the BM is directly related to the ability to hear a sound. When the BM is stimulated by the fluid pressure difference induced by the movement of the middle-ear (ME) structures (i.e., tympanic membrane, malleus, incus, and stapes), the hearing pathway is called air conduction (AC) [1]. On the other hand, when the BM is stimulated by vibration of the skull (or head), the hearing pathway is called bone conduction (BC). The mechanism of sound-energy transmission from the skull vibration to the BM motion is often explained by five contributors which are (1) inertia of the ME ossicles, (2) compression and expansion of the bony shell of the cochlea, (3) inertia of the cochlear fluid, (4) deformation of the ear canal, and (5) sound pressure transmission from the cerebrospinal fluid [2, 3]. However, the most important contributor for the BC driven BM vibration at different frequencies is still unclear.

To reveal the dominant contributor for the BC driven BM motion, the cochlea and the skull/head vibrations have been investigated through experiments as well as simulations. For example, in order to study the cochlea in BC hearing, the BM velocities in human temporal bone specimens were investigated when the stimulation was by BC [4]. Recently, Chhan et al. [5] measured fluid pressure of the chinchilla cochlea while manipulating the ME condition when stimulation was by BC. Through the measurement of the fluid pressure, they showed the significance of the cochlear fluid inertia or compression in BC hearing. In addition, there are also numerous experiments for investigating the skull/head vibrations in BC hearing. Stenfelt et al. [6], using a dry human skull, investigated the mechanical point impedance (**Z**_*m*_) and the acceleration response of the bone encapsulating the cochlea during BC stimulation at various positions on the skull. Furthermore, their study was extended to human cadaver heads [7], as well as live human skulls [8]. In this line of studies, the authors showed that there were differences in the resonance frequency of **Z**_*m*_ between the dry skull and cadaver and live human heads, and there were also differences between cochlear vibrations as measured in the dry skull and the cadaver and live human heads. However, the experimental work is limited in revealing the BC mechanism because it is difficult (1) to measure the cochlea or skull response with BC stimulation due to the vibrations of the whole specimen, (2) to measure the cochlear or skull response in a live human, and (3) to analyze the effect of a specific component of the cochlea or skull on the BC hearing due to the complex geometry and inaccessibility.

To partly overcome the above-mentioned limitations, finite-element (FE) models of the human cochlea and skull have been developed for numerical simulation of BC hearing. Kim et al. [9, 10] showed the significance of the antisymmetric pressure component in BC hearing using an FE model of the human cochlea and ME structures. While Kim et al. used inertia of the ME ossicles and cochlear fluid for the BC stimulation, Böhnke and Arnold [11] used compression and expansion of the bony shell of the cochlea by applying a dynamic pressure to the cochlear wall of the model. Their simulations showed the possibility of canceling a BC tone by an AC tone of the same frequency, similar to the famous experiment by von Békésy [12]. However, these studies are limited as only one factor, such as only inertia or only bone compression, is studied. In reality, more than two factors are combined for hearing of BC sound. In addition, the influence from the skull/head itself on BC hearing (e.g., sound transmission from the BC stimulation position to the cochlea) was not included. One way to overcome these limitations is to construct a whole head model. Such whole head models exist [13, 14]. However, most models were aimed at investigating the effects of the head size or the material properties on skull fracture and head injury rather than BC hearing. One exception is the model developed by Taschke and Hudde [15]. This was an FE model of the human head including the auditory periphery. Using that model, they showed the displacement and pressure distribution of the ME and the cochlea when stimulation is by BC. The limitations of that study are that (1) no validation of the model was reported and (2) the detailed information of each component of the model, such as mechanical properties, was not given.

Consequently, there is a need for a whole head model for investigations of BC sound. Therefore, a new FE model of a dry skull was constructed based on cryosectional images of a human female. For validation of the model, the **Z**_*m*_ of the skull and the acceleration of the cochlea were compared with experimental data in the literature. The model would further the understanding of BC sound transmission in the skull as well as vibrational pattern of the skull important for BC hearing.

## 2. Methods

The geometry of the model was obtained by 3D reconstruction of high resolution (0.33 × 0.33 × 0.33 mm) cryosectional color images of a human female. The images were obtained through the Visible Human Project (http://vhnet.nlm.nih.gov/).

### 2.1. FE Mesh and Mechanical Properties

**Figure 1:**
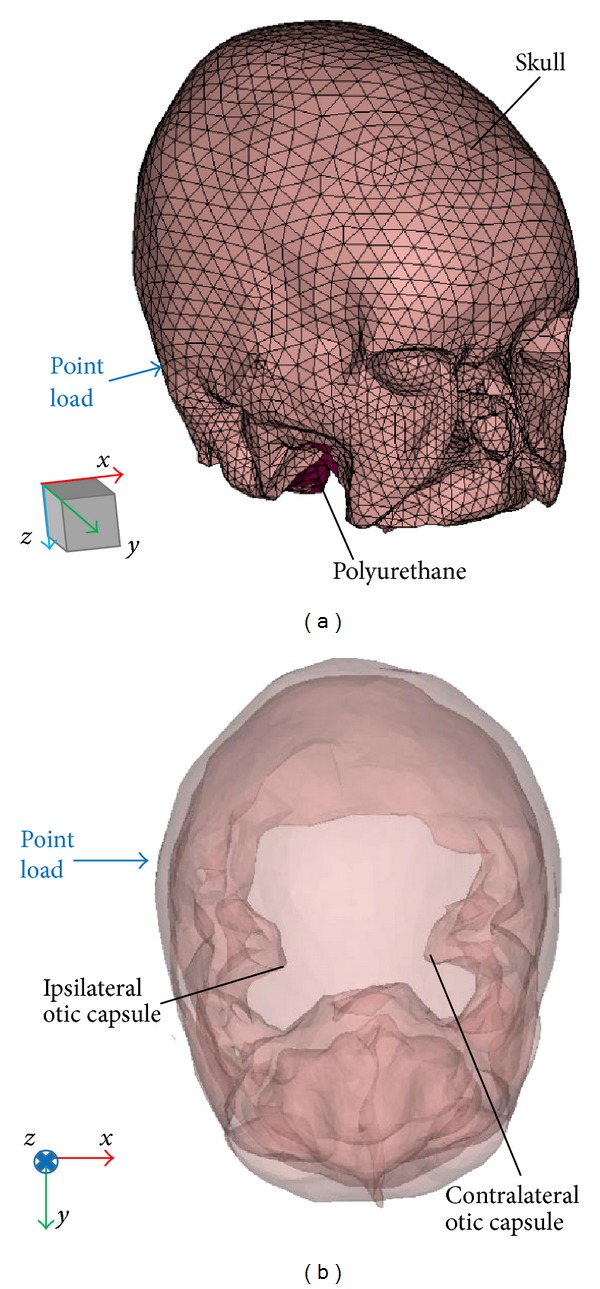
(a) The geometry of the model skull shown as finite-element meshes of the skull and polyurethane. (b) Top view of the skull model. The cranial vault and the attached polyurethane are here transparent to allow visualization of the cochlear bone.

An FE mesh of the model was created using the FE pre/postprocessing software HyperMesh (Altair Engineering, Troy, MI, USA). The *x*, *y*, and *z* directions of the model (rectangular coordinate system) were set to be the medial, anterior, and inferior directions of the skull, respectively (see Figure 1). This is in line with the coordinate system used for the experimental data in Stenfelt et al. [6].

According to Stenfelt et al. [6], 340 g of polyurethane was poured into the dry skull to increase the damping giving an approximately 5 mm thick layer of viscous-elastic damping material inside the skull. Therefore, to address more realistic conditions, polyurethane was also modeled in the FE skull model. The skull and the polyurethane were meshed with 32,000 and 18,000 four-noded tetrahedral solid elements, respectively. The mass of the bone and polyurethane was set to be 470 g and 340 g, respectively, for the consistency with that of the experimental settings.

The skull is composed of two layers of cortical bone (i.e., tables) separated by cancellous bone (i.e., diploë). Nevertheless, in this study, the skull was assumed homogenous for simplicity. This simplification was also used in the model of Taschke and Hudde [15] who studied the BC hearing mechanism. Previous studies [14, 16] reported Young's modulus of the tables and diploë in the skull of a normal human head to be 15 GPa and 4.6 GPa, respectively. Kanyanta and Ivankovic [17] reported Young's modulus of the polyurethane as 1 MPa. Based on these studies, the values for Young's modulus in the simulation were determined by tuning the resulting **Z**_*m*_ of the skull and cochlear acceleration. The values for the mechanical properties in the model are summarized in Table 1.

### 2.2. FE Analysis

The commercial FE software, ACTRAN (Free Field Technologies, Belgium), was used for the simulations. For the analysis of the forced responses of the skull from an external force, the following equation of the motion (EOM) was used:

(1) $$\begin{array}{c} K\cdot x-\omega^{2}M\cdot x=f, \end{array}$$

where *ω* is the angular frequency, **M** and **K** are the stiffness and mass matrices, respectively, and **x** is the displacement vector to be solved as a response to the force vector, **f**. The stiffness and damping properties related to the structural components are represented by the frequency-dependent complex-valued material modulus:

(2) E(ω)=E1(ω)+jE2(ω)=E1(ω)(1+jη(ω)),

where *E*_1_ is the “storage” modulus indicating the stiffness and *E*_2_ is the “loss” modulus representing the damping. The loss factor, *η*, indicates the material damping. In the current skull model, the *E*_2_ of the polyurethane is assumed to be frequency dependent. Therefore, in Table 1, the *η* of the skull has constant value, whereas the *η* of the polyurethane has frequency-dependent value. The following equation is used for the *η* of the polyurethane:

(3) $$\begin{array}{c} \eta \left(\omega \right)=\alpha \omega , \end{array}$$

where *α* is constant. The values of *η* for the polyurethane are 0.01, 0.1, and 1 at 0.1, 1, and 10 kHz, respectively.

The general FE formulations [18] are used to obtain the stiffness matrix, **K**, such as

(4) $$\begin{array}{c} K=\overset{}{\underset{e}{\sum }}\overset{}{\underset{V_{e}}{\int }}B^{T}\cdot D\cdot BdV, \end{array}$$

where *e* is the number of elements, *V*_*e*_ is a typical volume element, **B** is the strain-displacement matrix, and **D** is the matrix of differential operators that convert displacement to strain.

Consequently, the stiffness matrix, **K**, in (1) is complex-valued and depends on the frequency:

(5) K(ω)=K1(ω)+jK2(ω),

where **K**_1_ and **K**_2_ represent the overall stiffness and damping of the system, respectively.

### 2.3. Validation

The developed FE model was validated by comparing **Z**_*m*_ and acceleration of the cochlear bone with published experimental data in Stenfelt et al. [6]. In the FE simulation, the dynamic force was applied 35 mm behind the ear canal opening in the medial direction, that is, *x*-axis (Figure 1). This is consistent with position 2 reported in Stenfelt et al. [6]. **Z**_*m*_ was defined by dividing the applied force (**f**) by the velocity (**v**) (i.e., **Z**_*m*_ = **f**/**v**) at the point of the applied force. It should be noted that the point force in the simulation corresponds to the force applied on an approximate area of 3 mm in diameter in the experiment. The diameter, 3 mm, is similar to the size of the screw used for the experimental measurements. For the measurement of the cochleae acceleration, Stenfelt et al. [6] cemented an adapter at the arcuate eminence (top portion of the petrous part of the temporal bone). In this study, the acceleration was calculated at the nodes of the skull near the arcuate eminence with the assumption that the accelerations of the nodes in this area are similar to each other.

## 3. Results

The **Z**_*m*_ of the skull and the acceleration of the cochlear bone were calculated and compared with results in Stenfelt et al. [6]. Additionally, a parametric study was performed by varying the values of the mechanical properties of the structures.

### 3.1. Mechanical Point Impedance with Changing of Mechanical Properties of Polyurethane

**Figure 2:**
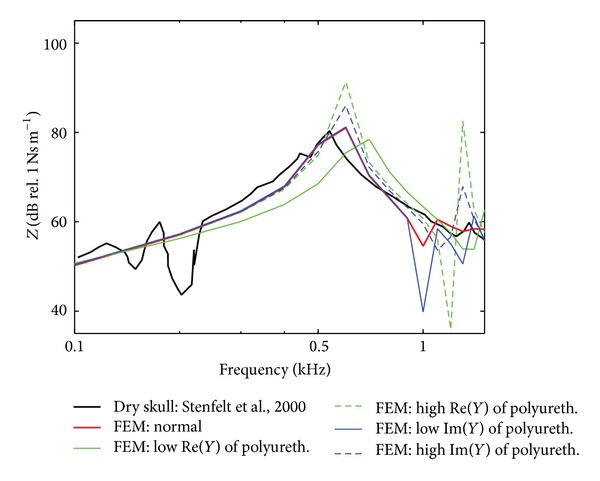
Level of the mechanical point impedance, **Z**_*m*_ = **f**/**v**, of the dry skull. The black-solid line represents the experimental data in Stenfelt et al. [6] and the solid red line (normal) is the results with the optimized values in the model. Young's modulus of the polyurethane was altered by increasing or decreasing its real (Re) or imaginary (Im) parts by two orders of magnitude. For example, complex Young's modulus, {*A* + *Bi*}, is {1*e*6 + 1*e*4*i*} for the “normal,” “high *Im*⁡(*Y*)” means {1*e*6 + 1*e*6*i*}.

Figure 2 shows **Z**_*m*_ of the model. Also included in Figure 2 is **Z**_*m*_ in Stenfelt et al. [6]. When Young's modulus of the bone and polyurethane was set to be 7.3 GPa and 1 MPa, respectively (red-solid line in Figure 2), the resonance frequency as well as the level of **Z**_*m*_ of the skull model was similar to the experimental data (black-solid line, [6]). The damping represented by the imaginary part of Young's modulus mainly affected the magnitude of **Z**_*m*_. This is indicated by the blue lines where the resonance frequency is unaltered in Figure 2 even if the magnitude of the imaginary value of polyurethane Young's modulus is changed. On the other hand, the stiffness, represented by the real part of Young's modulus, affected both the magnitude and resonance frequency of **Z**_*m*_. When the real part was increased from 1 MPa to 100 MPa (green-solid line in Figure 2), the magnitude of **Z**_*m*_ decreased 3-4 dB whereas the resonance frequency increased to 0.6-0.7 kHz. On the contrary, when the real part was decreased to 0.01 MPa (green-dotted line), the magnitude of **Z**_*m*_ increased 8-9 dB whereas the resonance frequency was nearly unchanged.

**Figure 3:**
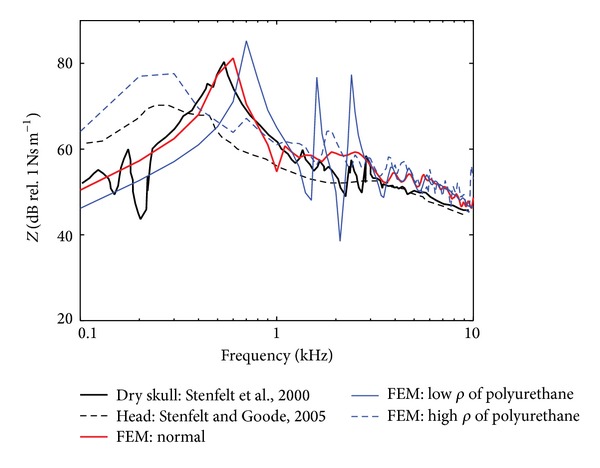
Level of the mechanical point impedance, **Z**_*m*_ = **f**/**v**, of the dry skull for three densities of the polyurethane. From the optimized value (997.40 kg/m^3^) in the model (represented by red-solid line and designated by “normal”), one order of magnitude was decreased to represent low density (i.e., 99.740 kg/m^3^). For the representation of the higher density, 8,800 kg/m^3^ was used for the density of the polyurethane to make the sum of mass of the skull and polyurethane be 3.47 kg. Also included in the figure is the level of the mechanical point impedance of the dry skull in Stenfelt et al. [6] (black-solid line) as well as the level of the mechanical point impedance from intact cadaver heads [7].

The effects of increasing or decreasing the density of the polyurethane on **Z**_*m*_ are shown in Figure 3. The optimized results (red-solid line) were obtained by 340 g of polyurethane. As expected by the general relationship between resonance frequency and mass (i.e., the resonance frequency is proportional to the inverse of square root of the mass), increasing the mass of the polyurethane (blue-dash line; 3 kg) lowers the resonance frequency, and vice versa (blue-solid line; 34 g). Specifically, when the mass is similar to that of human head (3 kg), **Z**_*m*_ of the dry skull model (blue-dash line) resembles that of a real human head, indicated by the black-dashed line (data taken from Stenfelt and Goode [7]).

### 3.2. Acceleration of the Ipsilateral and Contralateral Cochlear Bone

**Figure 4:**
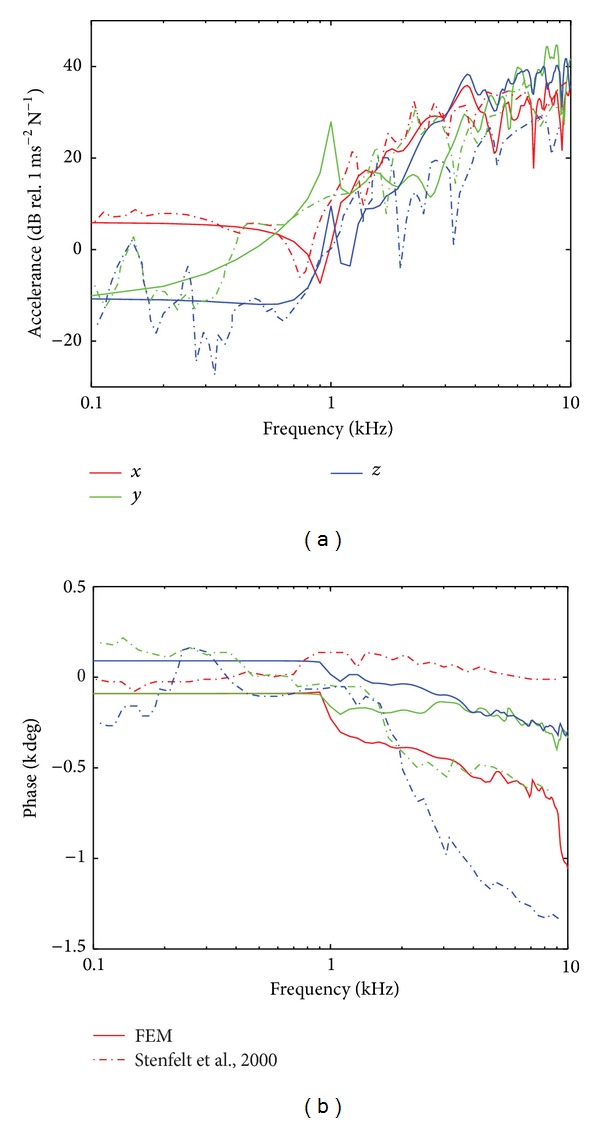
(a) Level (dB) and (b) phase (degrees) of the acceleration at the ipsilateral cochlear bone. In both (a) and (b), the red, green, and blue lines represent the *x* (medial-lateral), *y* (anterior-posterior), and *z* (inferior-superior) directional acceleration. In addition, solid lines indicate the results of the simulation while dashed-dotted lines show the results of the previous experiment.

**Figure 5:**
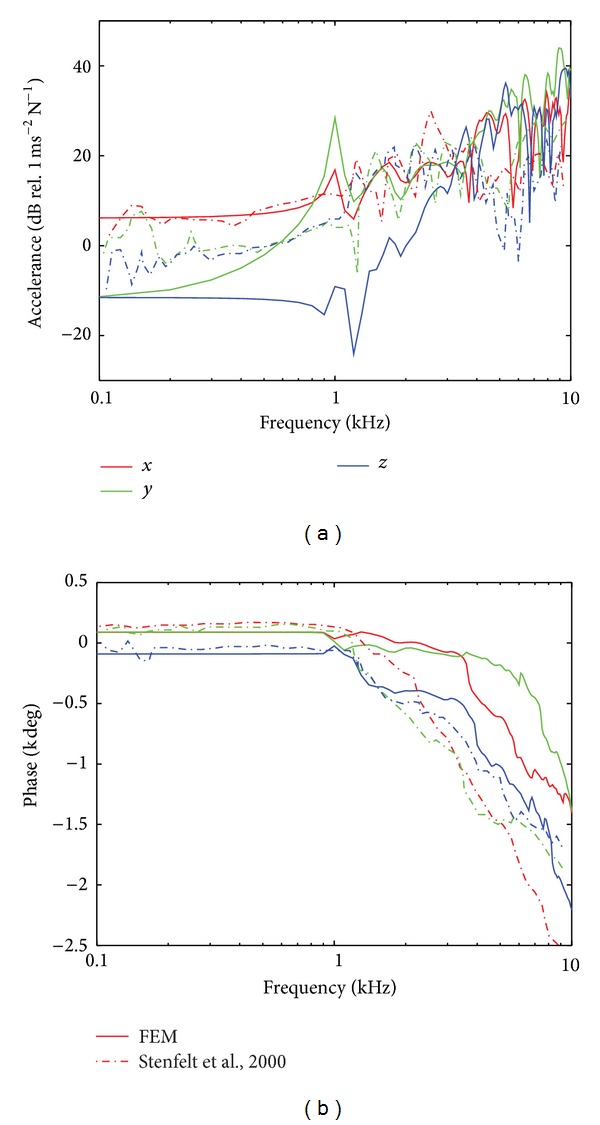
Same as Figure 4 but calculated in the contralateral cochlear bone.

The accelerations of the cochlear bone at the ipsilateral and contralateral sides of the model are shown in Figures 4 and 5. At both sides, the magnitude of the acceleration in the *x* direction (*a*_*x*_; medial-lateral direction) of the model was similar to that reported in the experimental study. The difference of the first antiresonance and resonance frequencies between the simulation and the experiment was about 100–200 Hz, whereas the magnitude difference of *a*_*x*_ was within 5–10 dB. Since the force was applied in the medial-lateral direction (i.e., *x* direction), the highest magnitude among the accelerations in the three different directions was observed in this direction. This can be the reason why the smallest discrepancy between the simulation and the experiment was observed in the *x* direction.

On the other hand, the magnitude of the *a*_*y*_ (anterior-posterior direction) and the *a*_*z*_ (inferior-superior direction) of the model showed larger discrepancies with those of the experiment. Specifically, the differences of the acceleration at the contralateral side are larger than those at the ipsilateral side.  Figure 4(a) shows the magnitude of the acceleration at the ipsilateral cochlea. Above 1 kHz, *a*_*y*_ showed 5–20 dB differences between the simulation and the experiment and *a*_*z*_ showed 5–30 dB differences. In Figure 5(a) showing the contralateral results, *a*_*y*_ and *a*_*z*_ showed differences of about 10–25 dB and 5–35 dB between the simulation and the experiment. In addition, while the differences of the acceleration were mainly observed above 1 kHz in the ipsilateral results (Figure 4(a)), the differences were observed for the whole frequency range, 0.1–10 kHz, in the contralateral results (Figure 5(a)). It should be noted that the greatest differences were seen when one of the traces, either the simulation or the experimental data, showed a resonance or an antiresonance. Consequently, these differences were of narrow frequency ranges.

For the phases shown in Figures 4(b) and 5(b), the simulation results (solid lines) at both ipsilateral and contralateral sides were consistent with the experimental results [6] up to 1 kHz. However, above 1 kHz at the ipsilateral cochlea (see Figure 4(b)), the phase of *a*_*y*_ and *a*_*z*_ in the experiment showed about 2 and 4 cycles roll-off from 1 kHz to 10 kHz. In contrast, the phase of *a*_*y*_ and *a*_*z*_ in the simulation showed little roll-off (about 1 cycle) from 1 kHz to 10 kHz. In addition, as shown in Figure 4(b), while the phase of the ipsilateral *a*_*x*_ in the experiment was almost constant from 1 kHz to 10 kHz, in the simulation it decreased about 3 cycles from 1 kHz to 10 kHz. These differences at frequencies above 1 kHz are mainly due to the resonances and antiresonances in the traces. For example, the simulated *a*_*x*_ shows a rapid roll-off at 1 kHz associated with the antiresonance at this frequency. The same antiresonance in the experimental data shows a phase lead and the difference between the experimental and simulated phase traces is around two cycles. However, the slopes of the two phase traces are nearly identical indicating the same BC wave transmission speed. Consequently, the difference in phases between the experimental and simulated BC cochlear responses is primarily due to the resonances appearing differently than general differences in structural responses.

At the contralateral side (Figure 5(b)), the phase of the experimental results for the *x* and *y* directions decreased more rapidly than the simulation results above 1 kHz. In the *z* direction, the acceleration of the cochlea shows reasonable consistency between the simulation and experiment above 1 kHz. The same argument of difference in resonances and antiresonances between the experimental and simulated responses can be made for the contralateral data as with the ipsilateral data.

**Figure 6:**
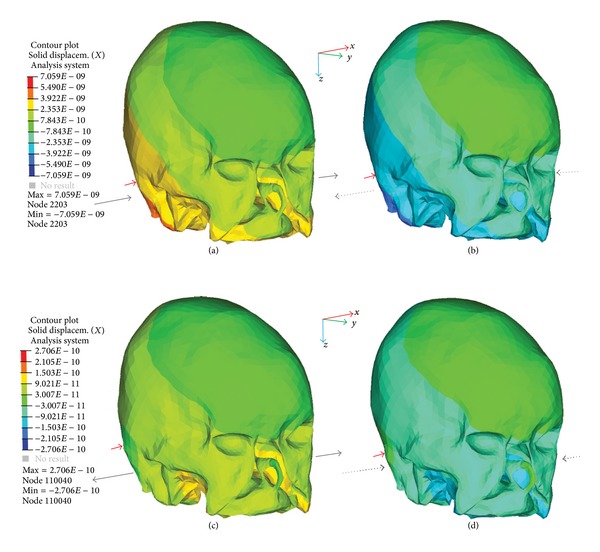
Contour plot of the *x* directional displacement of the skull. The same row and column represent the same simulated frequency and phase, respectively. The simulated frequencies are 100 Hz in (a) and (b) and 600 Hz in (c) and (d). The phase difference of the displacement between ((a) or (c)) and ((b) or (d)) is 180 degrees. Red arrows indicate the position and direction (i.e., *x*) of the applied force (1 *μ*N). Gray arrows with the same line type represent the movement of the skull at the ipsilateral and contralateral sides in the same phase. The skull shows the translational motion in (a) and (b), whereas the skull shows the contraction and expansion in (c) and (d). The legend for displacement in (a) and (c) corresponds to the simulations in the same row. For example, the legend in (a) covers (a) and (b). The “displacem.” in the legend means the displacement in millimeters (mm).

The *x* directional displacements of the skull at 100 Hz and 600 Hz are shown in Figure 6 in a contour plot. While the vibration of the skull was approximated as a rigid body motion at 100 Hz, a different mode shape was observed at 600 Hz. The motion at 600 Hz resembled contraction and expansion of the skull rather than the translational motion and the two sides of the skull moved with opposite phases. As the stimulation frequency increased, the numbers of modes of the skull increased. The increased number of modes can cause local rotational motion. Some of the discrepancy between the simulation and the experimental data could be caused by this local rotational motion.

## 4. Discussions

### 4.1. Mechanical Point Impedance of a Human Head

The mechanical point impedance (**Z**_*m*_) of a dry skull was investigated in order to tune the values of the mechanical properties of the bone and the polyurethane in the model. As shown in Figures 2 and 3, the optimized **Z**_*m*_ (red-solid line) showed the resonance frequency to be 600 Hz with a magnitude of 82 dB Ns/m, which was about 100 Hz and 2 dB different from the resonance frequency and the magnitude in Stenfelt et al. [6]. Franke [19] reported the resonance frequency of **Z**_*m*_ to be 500 Hz in a dry skull experiment. In his experiment, damping was added to the dry skull by pouring gelatin in the cranial space. McKnight et al. [20] also reported **Z**_*m*_ in human dry skull experiments. They observed the resonance frequency of **Z**_*m*_ of the dry skull at 680 Hz and 800 Hz when the mass of the dry skull was 652 g and 440 g, respectively. The stimulation in McKnight et al. [20] was applied 55 mm behind the ear canal in the posterior/superior direction. Since the mass of the dry skull and the force location in the previous studies [19, 20] are different from those of the current study, it is difficult to compare **Z**_*m*_ of the current study directly with the previous ones. However, the small discrepancy indicates that (1) there is a spread of skull geometry and mass and (2) **Z**_*m*_ of the current study is similar compared to other studies of dry skulls.

Based on the dry skull results, **Z**_*m*_ of a real human head can be estimated through the current FE model. According to Stenfelt and Goode [7], the masses of six human cadaver heads were reported to be between 3.25–3.78 kg. Therefore, we modified the mass of the polyurethane in the model to be 3 kg (i.e., sum of mass of skull and polyurethane is 3.47 kg), and then **Z**_*m*_ was calculated (blue-dash line in Figure 3). When we compared **Z**_*m*_ of the modeled 3.47 kg human head with the published data (black-dash line, [7]), the resonance frequencies of the two cases occurred at similar frequency ranges, 200–300 Hz. Also, there was about a 7 dB difference in the magnitude of the two **Z**_*m*_ at the resonance frequency with less difference further away from the resonance frequency. According to Figure 2, complex Young's modulus of the inner component (i.e., polyurethane in the current study) does not significantly affect the resonance frequency of **Z**_*m*_. Therefore, the calculated **Z**_*m*_ of the 3.47 kg human head can be reasonable since the assumed mass is close to that of a real human head, whereas assumed Young's modulus of the inner component can be different from that of a real human head. In other words, in the current human-head model, the consistency of the resonance frequency of **Z**_*m*_ in Figure 3 is more important than the inconsistency of the magnitude of **Z**_*m*_.

### 4.2. Acceleration of the Cochlear Bone

For frequencies below 600 Hz, the magnitude of the acceleration at the two cochleae is the greatest in the *x* direction. This means that the *x* directional vibration is the dominant direction below 600 Hz when the BC stimulation was applied 35 mm behind the ear canal opening. With the same stimulation position, however, the three orthogonal directions showed similar vibration responses at the ipsilateral cochlea at frequencies above 1 kHz and at the contralateral cochlea at frequencies above 4 kHz. In other words, at the higher frequencies, there was no directional effect from a specific stimulation direction of the structure. This was also found in the experimental studies of cochlear vibration during BC stimulation [6, 7].

Up to 1 kHz, the magnitude and phase of the acceleration of the ipsilateral and contralateral cochleae in all directions showed reasonable consistency between the simulation and the experiment [6] except the magnitude of the contralateral *a*_*z*_ (Figures 4 and 5). This indicates that the vibration pattern of the dry skull in this study is reliable at least up to 1 kHz in comparison with that in the experiment. Above 1 kHz, as discussed above, the phase differences increase in both ipsilateral and contralateral cochleae. However, the results can be meaningful when we consider the group time delay, *τ*_*gd*_, defined as

(6) $$\begin{array}{c} \tau_{gd}=-\frac{1}{2\pi }\frac{d\phi \left(f\right)}{df}, \end{array}$$

where *ϕ*(*f*) is the phase shift in radians and *f* is the frequency in Hz. The *τ*_*gd*_ of the simulation at all directions in both ipsilateral and contralateral cochlea is similar to that of the experiment except for *a*_*z*_ in the ipsilateral cochlea. This means that the wave speed through the dry skull and polyurethane of the FE model is comparable to that in the experiment.

The current model does not provide information of the different pathways important for BC hearing, such as the ear canal sound pressure or the fluid inertial effect inside the cochlea [2, 3]. However, since the model can provide the vibrational response of the skull, it can be useful for the BC excitation in the isolated 3D middle-ear and cochlear FE model [10]. The drawback of such isolated model is that the true excitation pattern of the surrounding bone during BC excitation is unknown. The currently presented model can provide such information. In other words, based on the current model, predictions of the proper BC excitation can be applied to the isolated 3D models. Furthermore, the current model can be used to predict the best position for BC hearing devices (e.g., BAHA, http://www.cochlear.com/; SoundBite, http://www.sonitusmedical.com/) because the simulation results of the model can indicate the position that produces maximum vibration at the cochlea for a specific frequency range. Another area where the model can further the understanding is the sensitivity of BC sound from a sound field [21]. Such simulation may reveal ways to improve the maximum attenuation from hearing protection devices.

## 5. Conclusions

A finite-element model of a human dry skull added with polyurethane was developed and analyzed to gain insight into the dynamic characteristics of a dry skull. The model shows mechanical point impedance and cochlear acceleration that is similar to experimental data in the literature. Although there are differences in the vibration characteristics between a dry skull and a human head, the simulated result from the dry skull can be helpful when analyzing an intact human head with proper adjustment of the parameter values. Moreover, the model may also be used to provide the input to an isolated middle-ear and cochlear model for BC sound.

## Figures and Tables

**Table 1:** Material properties of components in the FE model of the dry skull.

Component	Elastic modulus *E*_1_ (MPa)	Density *ρ* (kg/m^3^)	Poisson's ratio *υ*	Loss factor *η*
Skull	7,300	870.23	0.3	0.01 (constant)
Polyurethane	1	997.40	0.33	0.1 at 1 kHz
